# Pneumonia due to *Mycobacterium shimoidei*: a rare non-tuberculous mycobacterial infection in a young patient with anorexia nervosa

**DOI:** 10.1007/s15010-026-02795-x

**Published:** 2026-04-16

**Authors:** Ahmad Wael Sultan, Rolf Schwarzer, Martin Kuhns, Hildrun Haibel, Thomas Schneider, Rasmus Leistner

**Affiliations:** 1 Department of Gastroenterology, Infectious Diseases and Rheumatology, BIH Center for Regenerative Therapies, Charité Universitätsmedizin Berlin, Corporate Member of Freie Universität Berlin and Humboldt Universität Zu Berlin and the Berlin Institute of Health (BIH), Berlin, Germany; 2 Labor Berlin—Charité Vivantes GmbH, Berlin, Germany; 3 National and WHO Supranational Reference Laboratory for Mycobacteria, Research Center Borstel, Leibniz Lung Center, Borstel, Germany

**Keywords:** Mycobacterium shimoidei, Nontuberculous mycobacteria (NTM), Cavitary pulmonary disease, Next-generation sequencing (NGS), Anorexia nervosa, Antimicrobial therapy

## Abstract

**Background:**

*Mycobacterium shimoidei* is a rare, nontuberculous mycobacterium that predominantly causes pulmonary disease mimicking pulmonary tuberculosis. Fewer than 50 cases have been reported worldwide, with only two cases previously published from Germany. Known risk factors include structural lung disease and immunosuppression.

**Case presentation:**

A 37-year-old female patient with anorexia nervosa (BMI 14.5 kg/m^2^) and Gitelman syndrome, presented with general deterioration of condition and B symptoms. On admission, she displayed a high fever, hypotension and tachycardia. Chest imaging showed an inflamed large left upper lobe cavitary lesion and the patient was examined for *Mycobacterium tuberculosis* (tbc) pneumonia. Microscopy showed acid-fast bacilli but PCR was negative for tbc. The subsequent amplification of the gene for 16S RNA and its analysis by next generation sequencing (NGS) revealed *M. shimoidei*. Primarily based on literature research and later on antimicrobial susceptibility testing (AST), the patient was started on a combination therapy with Clarithromycin, Ethambutol and Rifabutin for a planned total of 12 months. She could be discharged after defeverescence and further clinical improvement but was regularly followed up as an outpatient. After terminating therapy, the patient showed complete radiological regression of pneumonia and clinical remission.

**Conclusion:**

This case adds to the limited literature on *Mycobacterium shimoidei* pulmonary disease and supports its role as a clinically relevant cause of cavitary nontuberculous mycobacterial infection. Severe anorexia nervosa may represent a predisposing condition as it might be associated with structural lung diseases. The case further underscores the importance of next-generation sequencing for the identification of rare NTM species.

## Introduction

*Mycobacterium shimoidei* is a rare non-tuberculous mycobacterium (NMT) that can lead to a severe pneumonia, especially in immunocompromised individuals [1, 2]. Due to its rarity, there is no clear consensus about its pathogenicity, risks factors and treatment. We report a case of a 37-year-old woman with severe anorexia and chronic kidney disease (CKD) based on the congenital Gitelmann syndrome who developed *M. shimoidei* pulmonary infection in Germany. This case highlights the diagnostic and therapeutic challenges regarding *M. shimoidei* infection.

### Case description

A 37-year-old woman from Germany was transferred from another hospital into our critical care unit for further evaluation and treatment of a sepsis with unknown focus. Initially the patient presented to the emergency room of an external hospital with unspecific symptoms like fatigue, coughing, abdominal pain radiating to the back, flu-like-illness and loss of appetite evolving for 5 days. She had a medical history of anorexia nervosa (BMI 14.5 kg/m^2^), multifactorial anemia, chronic kidney disease (CKD) based on a known Gitelmann syndrome (eGFR < 15 ml/min/1,73 m^2^). The latter was regularly controlled and treated with substitution of electrolytes but no need for dialysis. On admission, she displayed a high fever (39.0 °C), hypotension (90/50 mmHg), and tachycardia (133/min). Initial blood analysis in our hospital showed elevated inflammation markers, inflammatory syndrome, severe impairment of renal function, severe hypophosphatemia and anemia (Table 1).

**Table 1:** Laboratory findings obtained at the time of admission are summarized

Parameter	Reference Range	Value	Unit	Interpretation
Hematology				
Hemoglobin (Hb)	12.0–16.0 (f) / 13.5–17.5 (m)	7.5	g/dL	Decreased
Leukocytes	4.0–10.0	9.92	× 10⁹/L	Normal
Platelets	150–400	246	× 10⁹/L	Normal
MCV	80–100	101.4	fL	Increased
MCH	27–33	34.2	pg	Increased
MCHC	32–36	33.8	g/dL	Normal
Inflammation/Infection				
C-reactive protein (CRP)	< 5	39.1	mg/L	Increased
Procalcitonin (PCT)	< 0.05	1.56	ng/mL	Increased
Ferritin	30–400	735.8	µg/L	Increased
*Renal Function*				
Creatinine	0.6–1.3	4.59	mg/dL	Increased
eGFR	> 90	< 15	mL/min/1.73 m^2^	Decreased
Cystatin C	0.6–1.3	4.11	mg/L	Increased
*Liver Function*				
ALT	< 35–45	40	U/L	Normal / borderline
AST	< 35	49	U/L	Increased
*Other*				
Albumin	35–52	28.8	g/L	Decreased

**Fig. 1:**
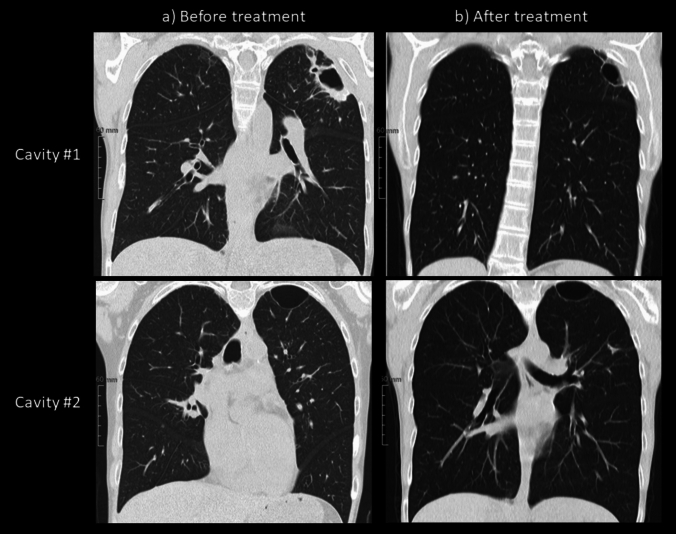
**A** Chest CT on admission showing two cavitary lesions in the left upper lobe. Only cavity #1 showed signs of inflammation. **B** Chest CT scan after 12 months of antimicrobial therapy. Cavity #1 showed complete regression of inflammation and significant reduction in size, whereas cavity #2 showed no relevant alteration

A chest CT imaging revealed multiple cavitary leasions in the left upper lobe. The patient had no history of smoking and no history of pulmonary disease. One cavity showed radiological signs of inflammation (Fig. 1a). Piperacillin and tazobactam was initiated as empirical treatment by the external hospital. In order to evaluate a suspected *M. tuberculosis* infection, the patient was transferred to our hospital and put under negative pressure contact isolation. Two sputum cultures 6 and 7 days after hospitalization revealed through microscopic examination acid-fast bacilli (auramine stain) with a negative PCR for *Mycobacterium tuberculosis* (tbc). However, further molecular analysis detected *Mycobacterium shimoidei* through next-generation sequencing performed directly on the primary specimen. Cultures were sent to the German Research Center Borstel (Leibniz Lung Center) to perform antimicrobial susceptibility testing (AST) (Table 1). According to these results and after dose adjustment for renal insufficiency and malnutrition, combination treatment with Clarithromycin 125 mg daily, Ethambutol 600 mg daily and Rifabutin 150 mg daily was initiated. However, in order to prevent dose related side effects caused by the significant renal insufficiency and the relevant malnutrition, clarithromycin was changed to moxifloxacin (400 mg once daily) after only 4 weeks. Under the initiated therapy, infection markers decreased, and the patient showed clear clinical improvement notably no further fever. Repeated sputum cultures after 10 and 15 days showed no further microscopic evidence of acid-fast bacilli. During an outpatient follow-up visit, based on the then available AST, we adjusted the antibiotic therapy, switched from clarithromycin to moxifloxacin due to the improved side effect profile and modified the medication dosages in accordance with the patient`s CKD.

The antimicrobial therapy was continued as a triple regimen consisting of moxifloxacin, rifabutin, and ethambutol for a total treatment duration of twelve months until October 2025. Regular clinical evaluations and laboratory monitoring were performed throughout the treatment course. Follow-up imaging, clinical examination and laboratory results showed no evidence of ongoing infection or disease activity. A control CT scan performed externally demonstrated a residual bulla in the left upper lobe without any signs of active inflammation or cavitary progression (Fig. 1b) (See Table 2).

**Table 2:** Phenotypic drug susceptibility testing performed by the National Reference Center for Mycobacteria in Borstel, Germany

Antimicrobial agent	MIC (mg/L)	Interpretation
Rifampicin	> 8	Resistant^®^
Rifabutin	1	Susceptible (S)
Macrolides (Azithromycin, Clarithromycin)	0,25	Susceptible (S)
Amikacin	≤ 1	Susceptible (S)
Doxycycline	16	Resista^®^(R)
Moxifloxacin	0,5	Susceptible (S)
Ciprofloxacin	4	Resi^®^nt (R)
Linezolid	≤ 1	Susceptible (S)
Trimethoprim/sulfamethoxazole	9,5	Susceptible (S)
Ethambutol	2	No interpretation available

Phenotypic drug susceptibility testing was performed using the microdilution method according to CLSI standards M24Ed2 and M62Ed1 (November 2018). *MIC* minimal inhibitory concentration. *R* resistant. *S* susceptible

## Discussion

Non-tuberculous mycobacteria (NTM) can cause pulmonary infections. Particularly individuals with structural pulmonary abnormalities or specific risk factors associated with a weakened immune system are at risk for NTM-associated pulmonary disease (NTM-PD) [3–5]. The pathogenesis is mostly a combination of environmental NTM exposure, pre-existing lung damage and host-specific immunological factors. Based on X-ray or CT images alone it is not possible to reliably distinguish between NTM-PD and pneumoniae caused by *Mycobacterium tuberculosis* (TBC-PD), thus diagnosis requires microbiological confirmation [3, 4].

Our patient initially showed acid-fast bacilli in her sputum, but tested negative for tuberculosis in a PCR test. The diagnosis of *M. shimoidei* was then made using next-generation sequencing (NGS) and was subsequently confirmed in bacterial culture at the German Reference Centre for Mycobacteria. After microscopy, conventional culture-based diagnostics of mycobacteria is time-consuming and usually takes several weeks, molecular methods like PCR or NGS can be significantly faster. PCR-based methods provide results within hours but is able to detect only a limited number of mycobacteria in question. NGS-based methods can detect virtually any mycobacterial species in an unbiased approach, still within a reasonable turnaround time of a few days) [6]. This shows that NGS can provide important information for timely decision-making in patient management even in sputum samples.

*M. shimodiei* was first described in 1975 by Tsakamuara et al. [7]**.** Since this initial description, fewer than 40 cases have been documented worldwide [1]. As the pathogen is difficult to identify, it is most likely underreported. The largest published series identified 23 cases in Queensland, Australia, in 2017 [1]. Most reported cases show patients with signs and symptoms of a pulmonary affection similar to those seen in other non-tuberculous mycobacterial infections, including cavitary lung disease and progressive respiratory disease [8]. Immunodeficiency and aspiration have also been described as risk factors in individual cases. There is no evidence in the medical literature of relevant extrapulmonary disease caused by *M. shimoidei*.

It has long been known and recognized that people with eating disorders can have a weakened immune system [9]. This is reversible and usually recovers after successful treatment of the eating disorder. However, the weakened immune system can promote infections with low virulence pathogens such as NTM. However, in our case a critical question arises regarding the role of *M. shimoidei* in pathogenesis of cavitary lesions. Does it serve as the primary agent causing cavitation or is it a secondary colonizer of an existing lung pathology, eventually causing infection? As the patient lacked known prior lung structural damage, severe anorexia nervosa must be discussed as a risk factor for developing cavitary disease [10]. The lungs are prone to adverse effects associated with anorexia nervosa or malnutrition in general. Several case reports have shown findings of emphysema in young patients with anorexia without history of smoking [11]. Two potentially life-threatening, though rare, complications are pneumothorax and pneumomediastinum. Spontaneous tension pneumoperitoneum and tension pneumothorax have also been described in patients with eating disorders who practice both restricted food intake and self-induced vomiting [12]. The association between low BMI and bulla formation is likely due to the reduced thickness of visceral fat in the pleura [13]. In the apical regions of the lungs, the visceral pleura is thinner than in the basal and caudal regions. This is likely one of the reasons why vesicles and bullae are frequently found in the lung apices. Our patient also showed multiple apical caverns but only few showed signs of inflammation. However, the cultural detection of living *M. shimoidei* and the clinical and radiological response to the antimicrobial therapy underscores an infection driven by *M. shimoidei* rather than a mere colonization. Ultimately, the most likely pathogenesis appears to be the development of multiple bullae associated with anorexia, which were secondarily colonized and eventually infected by *M. shimoidei*.

The patient also showed an additional illness, the so-called Gitelman syndrome. This is an autosomal recessive inherited renal tubulopathy [14]. Its primary effects are limited to renal electrolyte handling, resulting in hypokalemia, hypomagnesemia, metabolic alkalosis, and hypocalciuria. Patients with Gitelman syndrome do not exhibit primary or secondary immunodeficiency, nor do they have defects in macrophage or T-cell function, which are the main risk factors for mycobacterial infections. Therefore, it is very unlikely that the Gitelman syndrome played a pathophysiological role in this case or mycobacterial infections in general.

The therapy for nontuberculous NTM-PD is species-specific and drug susceptibility testing should guide therapy. The empirical treatment for NTM-PD includes a combination of a rifamycin derivative, ethambutol, and a macrolide [15]. The found *M. shimoidei* isolate in our case showed resistance to rifampicin but susceptibility towards rifabutin. In comparison to other NTMs, clinical data on *M. shimoidei* is exceptionally rare, and no formal guideline recommendations exist. Available case reports show rifampicin resistance in all cases, thus small series suggest a regimen of rifabutin, ethambutol and clarithromycin [1]. Other NTM species show variable rifampicin susceptibility patterns [15, 16]. Across NTM species, rifabutin consistently demonstrates lower MICs than rifampicin, with studies showing rifabutin has the most potent in vitro activity among rifamycin derivatives against many common NTMs. Treatment duration is at least 12 months after sputum culture conversion. In our patient's case, due to concerns regarding side effects, the therapy was changed after 1 month from clarithromycin to moxifloxacin. The initial therapy led to sputum culture conversion after 8 days and after regimen change and completion of in total 12 months therapy we observed complete clinical and laboratory remission.

## Conclusion

This case contributes to the limited evidence identifying *Mycobacterium shimoidei* as a clinically relevant cause of cavitary nontuberculous mycobacterial pulmonary disease. It supports the notion that *M. shimoidei* can act as a true pathogen rather than a mere colonizer. Severe anorexia nervosa may represent an underrecognized predisposing condition, potentially through the development of structural lung abnormalities. Furthermore, this case highlights the diagnostic value of next-generation sequencing for the timely identification of rare NTM species and demonstrates that individualized, susceptibility-guided combination therapy can lead to complete clinical and radiological remission, even in rare NTM infections.

## Data Availability

No datasets were generated or analysed during the current study.
